# M-Batches to Simulate Luminal and Mucosal Human Gut Microbial Ecosystems: A Case Study of the Effects of Coffee and Green Tea

**DOI:** 10.3390/microorganisms12020236

**Published:** 2024-01-23

**Authors:** Elizabeth Goya-Jorge, Irma Gonza, Caroline Douny, Marie-Louise Scippo, Véronique Delcenserie

**Affiliations:** 1 Laboratory of Food Quality Management, Department of Food Sciences, FARAH-Veterinary Public Health, University of Liège, B43b, 4000 Liège, Belgium; egoya@uliege.be (E.G.-J.); iegonza@uliege.be (I.G.); 2 Intestinal Regenerative Medicine Laboratory, Department of Clinical Sciences, College of Veterinary Medicine, North Carolina State University, Raleigh, NC 27606, USA; 3 Laboratory of Food Analysis, Department of Food Sciences, FARAH-Veterinary Public Health, University of Liège, B43b, 4000 Liège, Belgium; cdouny@uliege.be (C.D.); mlscippo@uliege.be (M.-L.S.)

**Keywords:** intestinal microbiota, human colon, batch fermentation, gut microbes, mucosa

## Abstract

Gastrointestinal simulations in vitro have only limited approaches to analyze the microbial communities inhabiting the mucosal compartment. Understanding and differentiating gut microbial ecosystems is crucial for a more comprehensive and accurate representation of the gut microbiome and its interactions with the host. Herein is suggested, in a short-term and static set-up (named “M-batches”), the analysis of mucosal and luminal populations of inhabitants of the human colon. After varying several parameters, such as the fermentation volume and the fecal inoculum (single or pool), only minor differences in microbial composition and metabolic production were identified. However, the pool created with feces from five donors and cultivated in a smaller volume (300 mL) seemed to provide a more stable luminal ecosystem. The study of commercially available coffee and green tea in the M-batches suggested some positive effects of these worldwide known beverages, including the increase in butyrate-producing bacteria and lactobacilli populations. We hope that this novel strategy can contribute to future advances in the study of intestinal ecosystems and host-microbe relationships and help elucidate roles of the microbiome in health and disease.

## 1. Introduction

Novel bioengineering approaches emerge every year to simulate the intestinal fermentation process, offering unique advantages and insights into the complex interactions in the microbial ecosystem of the human colon [1]. Batch culture or static colonic fermentation models are popular screening experiments conducted in individual bioreactors where the test substance is maintained in contact with microbial communities and basal media. These assays are generally performed for short durations, ranging from 24 to 48 h, with a maximum of 72 h. Several parameters can be controlled, including pH, temperature, and the maintenance of an anaerobic atmosphere [2,3].

Batch cultures offer several advantages, including their quick setup, cost-effectiveness, ease of operation, and reproducibility, thanks to their simple design [4]. Their high throughput and potential for automation make them an essential initial method for studying the composition, metabolism, and modulation of gut microbiota by probiotics and dietary compounds [5]. Moreover, these models can be easily downscaled, reducing the required media volume and concentration of tested compounds. Batch cultures are commonly employed as a preliminary step before conducting more complex experiments involving multi-vessel continuous fermentation [6]. The main disadvantages of the batch cultures are the rapid substrate depletion, the high accumulation of microbial metabolites, and the medium acidification, which prevents further microbial activity. The simulation of more complex gastrointestinal functionalities, such as peristalsis, is not possible in these models [4,7].

The mucosal compartment, which includes the mucus layer and the epithelial surface of the gastrointestinal tract, is a dynamic and essential environment that plays a significant role in shaping the behavior of the gut microbiota [8]. The main components of the mucus layer are mucins, which are constituted mainly by glycoproteins and oligosaccharides as side chains. The mucus layer acts as a barrier between the luminal contents (commensal and pathogenic bacteria, xenobiotic agents, and digestive enzymes) and the host epithelial and immune cells [9,10]. Some commensal gut bacteria, such as *Bacteroides*, *Parabacteroides*, *Bifidobacterium*, *Ruminococcus* members, or *Akkermansia muciniphila*, have the ability to degrade mucins present in the mucus layer [11]. Bacteria colonizing the mucus layer play an important role in modulating the host response to inflammation and the epithelial tight junction barrier [12]. It has been demonstrated that mucin supplementation induces significant increases in some mucin-degrading bacteria, such as *Bacteroides*, *Ruminococcus*, and *Akkermansia*, in long-term in vitro experiments [13]. Thus, incorporating microbial communities that inhabit the mucosal compartment in gastrointestinal simulations is crucial for a more comprehensive and accurate representation of the gut microbiome and its interactions with the host [14].

Although dynamic models have incorporated methods to simulate the mucus layer into colonic fermentation experiments [15], and interesting suggestions of adding mucins to porcine batch fermentation models are reported in the literature [16], human batch fermentations lack strategies as such. In this work, we aim to suggest M-batches as a novel approach to enhance the ecological relevance of these static and short-term fermentation models. As a case study, we simulated the fermentation of commercialized coffee and green tea and analyzed different setups of the experiment.

## 2. Materials and Methods

### 2.1. M-Batches Experiment

#### 2.1.1. Design of the Experiment

**Figure 1:**
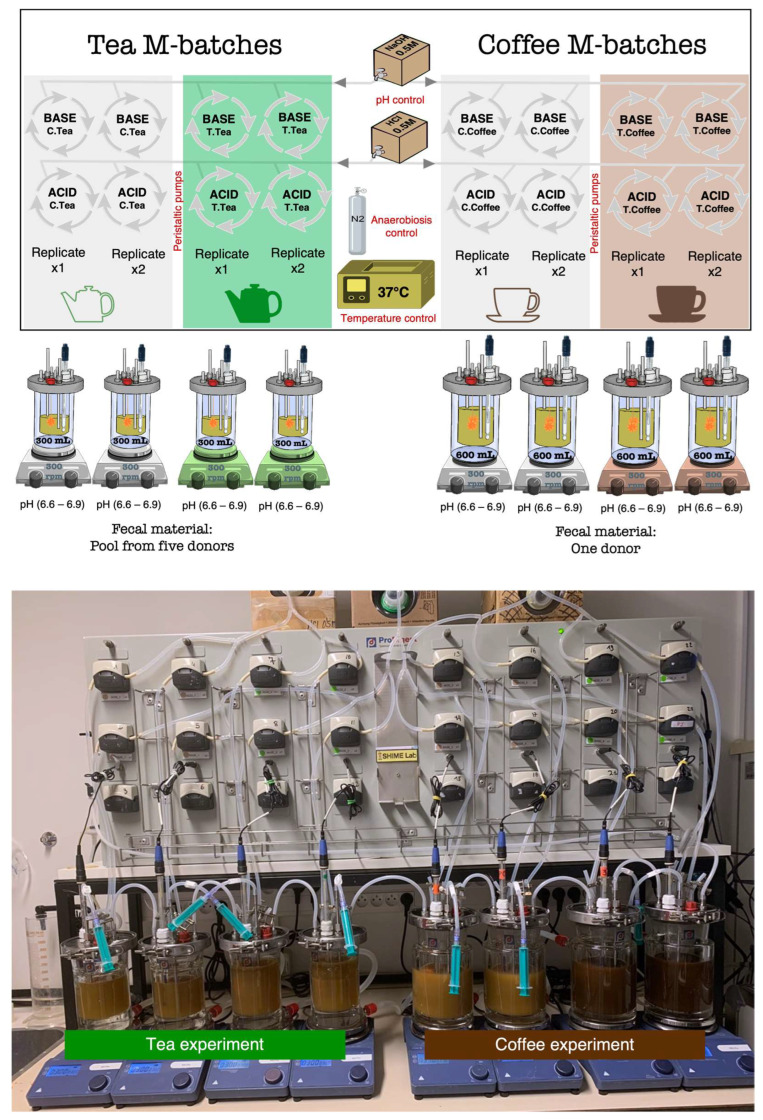
Set up of the M-batch experiments. The design is represented at the top, and a picture of the experiment is shown below.

As presented in Figure 1, two kinds of M-batches were designed. The first one, used to test the green tea, was assembled in small fermentation vessels with a maximum volume of 300 mL/vessel. The second one used to test black coffee was assembled in bigger vessels (600 mL maximum volume). The fecal inocula were also different (as detailed next). All conditions were repeated in independent experiments, and blank control fermentations were run in parallel to be compared to the treatments. Human body basal temperature (37 °C) and anaerobic conditions were maintained during all experiments that lasted 48 h. The pH was automatically maintained in a range from 6.6 to 6.9, as observed in the human adult descending colon, by peristaltic pumps that added acid (HCl-0.5 M) or base (NaOH-0.5 M) when needed. Treatments were inoculated together with the nutritional medium and the human gut microbiota samples (fecal inoculum). Mucin bags for simulation of the mucosal environment were tied to the vessels and submerged in the suspension mix that was maintained at constant agitation (300 rpm) using stirrers and magnetic beads.

#### 2.1.2. Human Stool Samples: Collection, Storage, and Inoculation

Collection and research with human fecal material were approved by the Ethical Committee of the Liège University Hospital (file number 2020/402). Six healthy donors, including five females (20, 23, 27, and 59 years old) and one male (20 years old), voluntarily participated in the study. The participants declared no consumption of probiotics, prebiotics, or antibiotics in the six months prior to feces collection. They all had a normal BMI (<25 kg/m^2^) and did not follow any particular restrictive diet. The participants declared that they had not undergone surgery or suffered from chronic or acute intestinal diseases.

Fecal samples were collected and short-term stored by the donors in anaerobic containers until delivery in the laboratory (maximum 3 h delay). A phosphate buffer saline (per liter of H_2_O: 8.8 g of K_2_HPO_4_, 6.8 g of KH_2_PO_4_, and 0.1 g of sodium thioglycolate) was added to the solid feces to prepare a 20% (*w*/*v*) suspension that was placed in a double-coated stomacher bag and homogenized for 10 min.

Two kinds of fecal inocula were prepared. The first one (A) pools equal proportions of feces from the five different donors below 30 years old, and the other one (B) uses the feces from the 59-year-old female volunteer. The inocula were sent for metagenetic analysis immediately. The rest of the filtered fecal homogenates were stored at −80 °C after adding glycerol as a cryoprotectant (20% *v*/*v*). The pool of feces (inoculum A) was used for the tea experiment, and the feces from the single donor (inoculum B) were used for the coffee experiment. For fermentation in the M-batches, A and B fecal inocula were added at 5% (*v*/*v*) to the vessels containing the adult M-SHIME nutritional medium (content detailed below).

#### 2.1.3. Chemicals, Media, and Treatments

- Adult M-SHIME medium: nutritional media for fermentation experiments were obtained from Prodigest (Ghent, Belgium). The composition of the pre-mixed powder (g/L) was: 1.2 g arabinogalactan, 2.0 g pectin, 0.5 g xylan, 0.4 g glucose, 3.0 g yeast extract, 1.0 g special peptone, 2.0 g mucin, 0.5 g L-cysteine-HCl, and 4.0 g starch. It was prepared in demineralized water and autoclaved prior to use.
- Green tea treatment: One dose (~60 mL) taken from a cup of tea (250 mL) prepared using one tea bag from Lipton brand (acquired in the supermarket) was added to vessels with a 300 mL final volume vessel (in duplicate). The same amount of water was added as a blank control (in duplicate).
- Coffee treatment: the equivalent of two ristretto cups (~50 mL) of coffee from the Nespresso brand, the Ispirazione Napoli variety (acquired in the supermarket), was added to vessels with a 600 mL final volume (in duplicate). The same amount of water was added as a blank control (in duplicate).

#### 2.1.4. Mucin-Covered Microcosms

Materials (i.e., carriers, bags, wire, zip ties) and mucin powder from the porcine stomach lining for the preparation of the mucin carrier beads were all obtained from Prodigest (Ghent, Belgium).

For the preparation of coated microcosms (carrier beads), a previously proposed methodology was followed with some modifications [15]. Briefly, agar technical No. 2 from Oxoid (Basingstoke, UK) was added to warm (70 °C) water in constant agitation. Approximately 1 g of agar per every 100 mL of water was necessary to reach proper consistency, to which 350 μL of NaOH (10 M) and 5 g of mucin powder were added. The mix was boiled three times and cooled down for two-minute intervals at room temperature. Then, the mix was placed in 50 mL falcon tubes containing the carrier beads that, once shocked in the solution, were carefully taken out to solidify using sterile materials and an environment (under the laminar hood). Net bags containing 15 carrier beads each were filled and zip-tied, and a plastic wire was used to hang them from the vessel’s lids. The maximum storage time for the prepared mucin-covered carriers was tested to be 24–48 h (at 4 °C), after which the consistency is compromised. In the herein experiments, the carriers were prepared on the same day of inoculation.

### 2.2. Analysis of Microbial-Derived Metabolites

#### 2.2.1. SCFA by SPME-GC-MS

An optimized method from a previously validated technique [17] was used for the quantification in the fermentation samples of short-chain fatty acids (i.e., acetic acid (C2), propionic acid (C3), and butyric acid (C4)) and branched-chain fatty acids (i.e., isobutyric acid (iC4) and isovaleric acid (iC5)). The limits of quantification were: 2.0–99.90 mM (C2), 0.97–48.60 mM (C3), 0.57–28.37 mM (C4), and 0.16–7.94 mM (iC4) and 0.10–4.90 mM (iC5).

Briefly, a solid-phase microextraction (SPME) followed by gas chromatography coupled to mass spectrometry (GC-MS) was used. The detailed preparation of the fermentation samples and the chromatographic conditions were described previously [18].

#### 2.2.2. Ammonia and Biogenic Amines by UPLC-FLD

Using an adaptation of a method previously validated for the analysis of biogenic amines in meat samples [19], the following metabolites were quantified in the fermentation supernatant of the M-batches experiment: ammonia, 2-phenylethylamine, tyramine, putrescine, and cadaverine. The limits of quantification were: 0.235–32.883 mM for ammonia, 0.013–0.990 mM for 2-phenylethylamine, 0.015–1.752 mM for tyramine, 0.018–1.364 mM for putrescine, and 0.005–1.022 mM for cadaverine.

Briefly, ultra-performance liquid chromatography with fluorescence detection (UPLC-FLD) from Waters Corporation (Milford, MA, USA) was used, with an Acquity UPLC BEH C18 (2.1 × 100 mm, 1.7 μm) column and a UPLC BEH C18 VanGuard pre-column (2.1 × 5 mm, 1.7 μm). Fermentation samples (100 μL) were prepared by adding 25 μL of a 100 ng/μL solution of 1,7-diaminoheptane (internal standard) prepared in trichloroacetic acid (5%). Then, 475 μL of perchloric acid (0.4 M) were added, and the mixture was vortexed for 20 s and centrifuged (17,746× *g*, 5 min, at room temperature). This extraction was performed twice, and both supernatants were combined. One milliliter was then transferred to a 15 mL tube, where 200 μL of NaOH (2 N) and 300 μL of saturated NaHCO_3_ were added, with vortexing after each addition. The dansylation was performed by adding 2 mL of dansyl chloride (10 mg/mL in acetone) and incubating at 70 °C for 15 min. Excess of dansyl chloride was bonded to 100 μL of glycine (150 mg/mL in water), followed by a second incubation (70 °C, 15 min). Finally, the tube was centrifuged (5 min, 3700× *g* at room temperature), and 500 μL of the solution were poured into an injection vial, which was capped. Samples were kept at 5 °C in the autosampler until analysis. For the analysis, 5 μL were injected into the UPLC.

### 2.3. Analysis of Colonic Bacterial Populations

The 16S amplicon sequencing and microbiota profiling of the two fecal inocula (A and B) used in this work followed the same methodology that was detailed previously, and the same applies to the qPCR analysis of targeted bacterial populations performed in this study [18]. For the 16S rRNA gene profiling, the V1-V3 regions were targeted. The PCR primers F: -5′-GAGAGTTTGATYMTGGCTCAG-3′ and R: -5′-ACCGCGGCTGCTGGCAC-3′ were used for library preparation with added Illumina adapter overhang nucleotide sequences. PCR products were purified using Agencourt AMPure XP beads (Beckman Coulter, Brea, CA, USA) and submitted to a second PCR amplification using the Nextera XT Index kit v2 (Illumina Inc., San Diego, CA, USA). A second clean-up for PCR amplicons was carried out. Library quantification was performed using the Quant-iT™ dsDNA assay kit (Invitrogen™, Waltham, MA, USA), and PCR products were normalized to 10 ng/µL. A quantitative PCR (qPCR) was conducted using the KAPA SYBR^®^ FAST qPCR kit (KAPA BIO, Boston, MA, USA). Quantified libraries were pooled, denatured, and diluted to a final concentration of 6 pM with 30% Phix (Illumina) as internal control. Samples were sequenced using the MiSeq reagent kit v3 in the Illumina MiSeq system. Mothur v1.47 and VSearch tools were used for alignment, clustering, and chimera removal. Sequences were clustered in Operational Taxonomic Units (OTU) and compared to the SILVA v1.38 database for classification.

Several bacterial taxa related to health were analyzed using qPCR. The qPCR assays were performed using the CFX96 touch real-time PCR detection system (Bio-Rad Laboratories N.V., Temse, Belgium) associated with CFX Maestro software (version 4.1, Bio-Rad) for data analysis. The qPCR mixture consisted of 10 µL of Takyon^TM^ No Rox SYBR^®^ 2X master mix (Eurogentec S.A., Seraing, Belgium), forward and reverse primers at the right concentration (ranging from 150 nm to 500 nm), 2.5 µL of DNA template (10 ng), and the corresponding volume of molecular water to reach a total volume of 20 µL per reaction. References to the primer sequences used and the qPCR protocols validated with the corresponding primer concentrations and temperatures of annealing (Tann) are summarized in Supplementary Table S1. Results of qPCR are presented as relative quantification through the 2^−∆∆Cq^ method [20], relative to samples from Day 0 (inoculation), and normalized based on the quantification of the reference sequence used as total bacteria.

### 2.4. Cell-Based Analysis of AhR Induction

AhR_HT29-lucia™ cells were obtained from Invivogen (Toulouse, France) and maintained as suggested by the providers in the Growth Medium: DMEM, 4.5 g/L glucose, 2 mM L-glutamine, 10% (*v*/*v*) heat-inactivated fetal bovine serum (FBS), Penicillin-Streptomycin (100 U/mL–100 μg/mL), 100 μg/mL Normocin™, 100 μg/mL Zeocin™. For AhR reporter gene assays, Assay Medium was used as follows: DMEM, 4.5 g/L glucose, 2 mM L-glutamine, and 10% FBS. Culture conditions were 37 °C and humidified atmosphere (5% CO_2_) incubation.

For measuring the AhR induction caused by fermentation supernatants from the M-batches, cells were seeded at 2.5 × 10^5^ cells/mL in clear 96-well plates (100 μL/well) and incubated overnight to allow attachment. Confluence (80–90%) was verified under the microscope, and treatments (20 μL of filtered fermentation sample + 80 μL of cell assay medium) were added to complete a final volume of 200 μL/well. The blank controls used included cells treated with the Assay Medium only and cells treated with the fermentation’s nutritional medium (M-SHIME medium). A positive control of AhR activation (FICZ) was included in all plates.

## 3. Results

### 3.1. Chromatographic Quantification of Metabolites

In general, metabolite quantification did not show significant differences between the control and treatment groups, nor did the tea or coffee experiments. However, at the end of the fermentation period (48 h), a greater production of ammonia and most biogenic amines was observed in the fermenters corresponding to the tea experiment (Table 1). In fact, the biogenic amines 2-phenylethylamine, tyramine, and putrescine were detected in the tea experiment only. Furthermore, cadaverine concentrations in tea fermenters were, on average, 30 times higher than those observed in coffee fermenters. Finally, ammonia concentrations were also higher in the tea fermenters compared with coffee experiment, although to a lesser extent than cadaverine.

**Figure 2:**
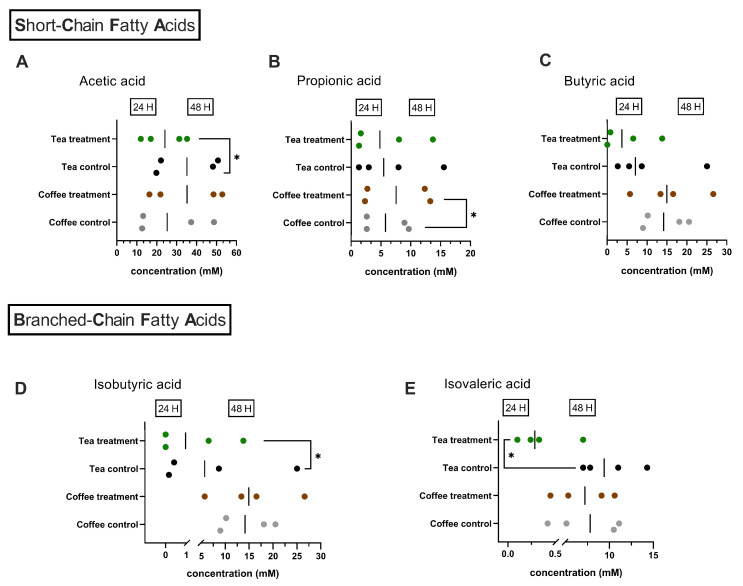
Production of Short-Chain Fatty acids and Branched-Chain Fatty acids in the M-batches experiment. Results represent individual measurements of the experiments conducted in duplicate and after 24 h and 48 h of fermentation. (**A**) C2: acetic acid; (**B**) C3: propionic acid; (**C**) C4: butyric acid; (**D**) iC4: isobutyric acid; (**E**) iC5: isovaleric acid. **Green dots**: treatment experiment (adding tea), **black dots**: control experiment (no tea added), **brown dots**: treatment experiment (adding coffee), **gray dots**: control experiment (no coffee added).

The production of SCFA was quantified after 24 and 48 h of fermentation. Similar concentrations of total SCFA were observed for both systems after 24 h and 48 h of fermentation. When analyzed individually, some significant differences were identified, as shown in Figure 2. When tea was added as a treatment, lower concentrations (*p* = 0.0202) of acetic acid were observed after 48 h of fermentation (Figure 2A). Coffee treatment caused a significantly greater (*p* = 0.0261) production of propionate after 48 h of fermentation (Figure 2B). No significant differences in butyrate production were observed with either tea or coffee when compared with the respective control experiments (Figure 2C).

Meanwhile, only with the addition of tea were significant differences in BCFA production observed. Thus, after 24 h of fermentation, lower production (*p* = 0.0194) of isovaleric acid was detected (Figure 2E), and after 48 h, lower amounts (*p* = 0.0129) of isobutyric acid were quantified in the microbiota exposed to green tea (Figure 2D).

### 3.2. Evolution of Colon Bacterial Populations

#### 3.2.1. Metataxonomic Analysis of the Fecal Inocula

**Figure 3:**
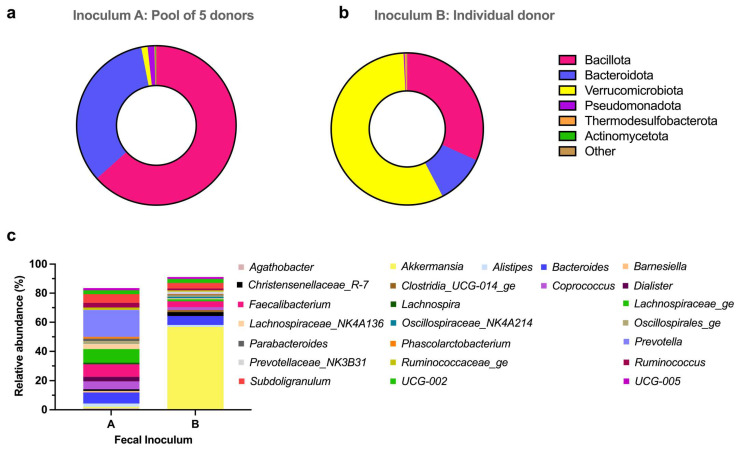
Relative abundances of the microbial communities in the fecal inocula used to perform the experiments. Phyla abundances in the fecal inocula in (**a**) The pool of five donors used for the tea experiment and (**b**) The feces from a single donor used for the coffee experiment. (**c**) The top 25 of the main genera identified in the two fecal inocula.

The 16S RNA sequencing of both fecal inocula used to perform the fermentation experiments yielded 10,000 reads per sample. At the phylum level, important differences were observed between the pool of fecal samples (inoculum A) used for the tea experiment and the individual donor feces (inoculum B) used for the coffee experiment. However, a similar frequency of the total counting of phyla identified by 16S rRNA Gene Sequencing was observed (Supplementary Figure S1). Bacillota and Bacteroidota were the major phyla present in inoculum A. Together, these phyla covered 96% of the total bacteria identified in this sample (Figure 3a). However, in inoculum B, the most abundant phylum was Verrucomicrobiota, which had a 56.83% prevalence. Bacillota (31.71%) and Bacteroidota (10.52%) were the other main phyla present in the sample from the single donor (Figure 3b).

The total number of genera identified in each inoculum was only slightly different. Thus, in the pool of feces, 128 genera were identified, while in the individual donor, 119 genera were counted. Nevertheless, the composition of the fecal inocula at the genus level showed important differences, as shown in Figure 3c. The main difference was the abundance of the *Akkermansia* genus. While in the pool of feces, this genus only represented 0.94% of the total bacteria identified, *Akkermansia* was the most abundant genus observed within the single donor, accounting for 56.82%. In the pool of feces, the most prevalent genera were *Prevotella* (18.5%), *Bacteroides* (7.53%), and *Alistipes* (2.27%) among the Bacteroidota phylum. The Bacillota phylum was mainly represented by the *Faecalibacterium* (8.38%), *Subdoligranulum* (5.99%), *Coprococcus* (5.44%), *Lachnospiraceae_NK4A136* (3.68%), and *Ruminococcus* (3.21%) genera. In the single donor inoculum, after *Akkermansia*, the second most abundant genus was *Bacteroides* (6.21%). Next, and within the Bacillota phylum, the genera *Faecalibacterium* (4.05%) and *Subdoligranulum* (3.84) showed the highest abundance.

Detailed 16S RNA sequencing data down to the genus level is provided as Supplementary Material for inoculum A (Table S2) and inoculum B (Table S3).

#### 3.2.2. qPCR Results

**Figure 4:**
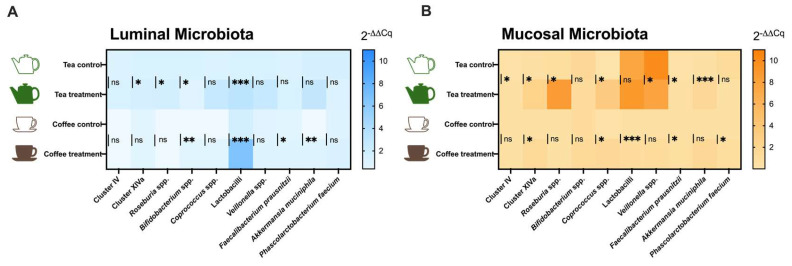
Relative quantification (2^−ΔΔCq^) of target bacteria populations measured by qPCR in the (**A**) Luminal and (**B**) Mucosal ecosystems of the M-batches. Mean values (n = 6) are shown as heat maps, and significant differences when compared to control vs. treatment are presented as ns: no significant (*p* > 0.05), * (*p* ≤ 0.05), ** (*p* ≤ 0.01), *** (*p* ≤ 0.001). Statistical comparisons were conducted using the unpaired T-test or the Mann–Whitney test, depending on the normality of the data.

The effects of tea and coffee consumption on the human gut microbiota were evaluated by qPCR analysis in the luminal (Figure 4A) and mucosal (Figure 4B) environments after 48h of fermentation. To this end, ten bacterial taxa generally reported in the analysis of gut microbiota and whose abundance is related to a healthy status were targeted.

The addition of green tea to the fermenters had a positive impact on butyrate-producing bacteria when compared with the control experiment. Thus, green tea better maintained the *Clostridium* cluster IV group in the mucosal environment, along with a slight increase in the relative quantity of *Clostridium* cluster XIVa in both luminal and mucosal ecosystems. Consistently, *Roseburia* genus, an important member of cluster XIVa, was also increased with tea addition in luminal and mucosal environments. The evolution of *F. prausnitzii* (belonging to cluster IV) showed that this bacterium was highly boosted in the mucosal microbiota when tea was added to the fermenters compared to the control group. The *Coprococcus* genus, which also includes some butyrate-producing species, was increased in the mucosal environment with tea as treatment. Lactic acid bacteria (LAB), such as Lactobacilli and *Bifidobacterium*, behaved differently. While a high increase in lactobacilli group was observed after tea addition in the luminal compartment, a slight decrease in the relative amount of *Bifidobacterium* genus was observed after the addition of tea. In the mucosal compartment, no differences from the control were observed in LAB in the case of the tea experiment. The relative quantity of *Veillonella* genus, a propionate producer group, reached high values in the control group of the tea mucosal environment. So, the increases observed with tea addition remained significantly lower than in the control group. Finally, the evolution of the mucin-degrading bacteria *Akkermansia muciniphila* was also monitored, and increased levels of this bacterium in the mucosal microbiota with tea as treatment were observed.

Meanwhile, the addition of coffee also caused a slight increase in butyrate producers, particularly in the mucosal environment, where cluster XIVa group, *Coprococcus* genus, and *F. prausnitzii* were found to be boosted. In addition, in the luminal microbiota, coffee favored an increase in *F. prausnitzii*. Interestingly, coffee caused significantly higher levels of *Bifidobacterium* and Lactobacilli in the luminal environment compared to the control experiment. In the mucosal environment, the lactobacilli group was also boosted by coffee. Better preservation of *A. muciniphila* in the luminal microbiota was observed when coffee was added to the system. Additionally, the preservation in the mucosal environment of *Phascolarctobacterium* faecium was facilitated by coffee.

### 3.3. Induction of AhR Activation Caused by the Metabolic Output of the M-Batches

**Figure 5:**
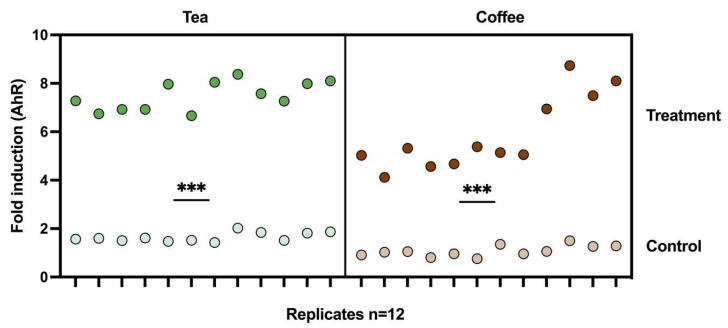
Fold induction of AhR transcriptional activation caused by the fermentation supernatant of the M-Batch experiments and relative to cells treated with the M-SHIME medium only. Individual values of technical triplicates for the duplicated experiments in two independent repetitions of the reporter gene assay (n = 12) are presented. Mean values of the treatments with coffee and green tea were compared with the control experiments, respectively, and the results were significantly higher in both cases (*p* ≤ 0.001) (***). **Green dots**: samples from tea experiment. **Brown dots**: samples from coffee experiment. Dark colors represent where treatment was added (tea or coffee) and light colors represent control experiments (no treatment added).

Compared with the control experiments, both tea and coffee treatments induced a significant activation of the AhR transcriptional pathway, as shown in Figure 5. Metabolic output from the tea seemed to have a slightly higher effect on AhR than coffee’s. However, this was also the case for the control experiments, where slightly higher values were obtained in tea’s control experiment compared to coffee’s control experiment.

## 4. Discussion

Dynamic gut models are reported in the literature, where niches for surface-attached microbes have been used to simulate mucin-adhered microbes [15,21]. In this work, we aimed to extrapolate the use of microcosm carriers to short-term fermentation in batches to simulate the luminal and mucosal human gut ecosystems, which we called “M-batches”. A similar suggestion has been previously reported in simulations of the porcine gut microbiota [16].

Among the known disadvantages of short-term and non-dynamic experiments are the limited nutrient availability and accumulation of metabolic waste products [3,5]. We limited this study to 48 h due to our previous experiences that showed us that 24 h was sufficient to reach a relative stabilization of the microbial communities present in the batch systems and that not many differences are commonly observed when compared to 48 h and 72 h in terms of metabolic production and microbial content [22]. Most measured parameters were therefore analyzed exclusively at the end of the fermentation period, except for quality control metabolites such as SCFA.

Batch experiments have been designed and reported in multiple set-up arrangements that in many cases depend on the nutritional medium, the adjustment of the pH, and, probably most importantly, the tested substances (e.g., prebiotics, probiotics, pharmaceutical formulations, food components) [4]. One of the most important variables is pH since it regulates microbial growth, enzymatic activity, and metabolic activity [23]. In this work, the pH was adjusted to the human descending colon (6.6–6.9) as it represents the final stage of the digestive process, and therefore the microbial populations of this colon section are the most represented ones in the feces.

The volume reported in batch experiments is very variable, ranging from deep-well plates, closed test tubes, or flasks to bioreactors of 70 mL volume [24] to 600 mL [18]. In our case, we investigated two different volumes (300 mL and 600 mL), and the different volumes seemed to influence the metabolic production and the microbial communities favored during the fermentation. For example, in the case of the biogenic amines and ammonia, significant differences were identified between the control experiment of tea (300 mL fermenters) and the control experiment of coffee (600 mL fermenters). Thus, these byproducts of amino acid fermentations accumulated more in the smaller vessels. However, as discussed below, other variables are likely influencing these observed differences. On the contrary, the concentrations of butyrate and its structural isomer isobutyrate detected in the control experiments were much higher in the case of higher-volume vessels (coffee experiment). The rest of SCFA and BCFA were similarly quantified regardless of the volume of the fermenters.

Another debated parameter is the preparation of the highest physiologically relevant fecal inocula [25] and the use of one or the combination of several feces to achieve a more representative colonic microbiota [26]. Pooling feces, particularly if more than four donors are used, does not seem to affect significantly the microbiota composition and, for batch experiments, has been recommended in standardized protocols [5]. For comparison, herein was designed one of the experiments with a single donor feces and the other one with a combination of 5-donor feces.

Indeed, the different inocula used in these two experiments could also play an important role in the different metabolites produced and the microbial composition. As observed in the metataxonomic analysis (Figure 3), substantial differences were identified at both phylum and genus levels when comparing the pool of feces (used for the tea experiment) and the single donor feces (used for the coffee experiment). Furthermore, when analyzing the evolution of the targeted bacteria in the control experiments, it was observed that the inoculum containing the pool of five donors seemed to provide a more stable luminal ecosystem. We draw this conclusion since the bacteria levels at the end of the fermentation period remained similar to the ones observed at the onset of the fermentation period. However, this was not the case in the control group of the coffee experiment using the inoculum from a single donor. In this system, most of the quantified bacteria decreased at the end of the fermentation period, indicating possible poor colonization of the fermenters. For the mucosal environment, we analyzed cluster XIVa, *Roseburia*, and *A. muciniphila* as colonization indicators since previous studies showed that these bacteria colonize mucins [21]. In this case, at the end of the fermentation period, in both systems, we observed a similar reduction in the relative amount of *A. muciniphila* in the control groups. The evolution of the other markers, cluster XIVa and *Roseburia*, was also similar in both systems. This may lead to the hypothesis that the origin of the microbiota was not necessarily the main factor determining the colonization of the mucosal compartment, since the inoculum from the single donor had a higher abundance of *A. muciniphila* (56.82%).

The use of mucin beads allowed better implantation of *Lactobacillus* in the mucosal environment of the 300 mL vessels containing the pool of feces of five donors, in agreement with previous reports [15]. The enrichment of *Lactobacillus* could explain the higher biogenic amine concentrations observed with the smaller volumes (300 mL) since *Lactobacillus* has been correlated with cadaverine, putrescine, tyramine, and tryptamine production [27]. However, in contrast with other studies [13], mucin supplementation did not have significant effects on *Akkermansia muciniphila* abundance, even when the microbiota used in the coffee experiment was highly rich in this bacterium.

Overall, both fermentation volumes and the used inocula allowed microbial growth in luminal and mucosal compartments. However, the pool of feces set up in the 300 mL vessels appeared to be moderately better at stabilizing some relevant populations, while some important metabolites like butyrate were better detected in the 600 mL vessels experiment. Therefore, this work provides additional evidence for the suitability of using a pool of feces to achieve a more standardized microbiota, particularly in cases where a predominant abundance of one genus (in this case, *Akkermansia*) is observed.

Regarding the impact of coffee and green tea, several studies have associated some of their health benefits with their effects on the human gut microbiome [28,29]. Consistently, through the M-batches experiments developed herein, the equivalent of two small cups of strong coffee and one average cup of tea did not cause dramatic effects in the metabolic production of SCFA, ammonia, and biogenic amines, indicating little disturbance of the gut microbiota. Moreover, both coffee and tea favored the growth of butyrate-producing bacteria and lactobacilli populations. In other studies, strong correlations have been reported between methylxanthines such as caffeine and theobromine (present in both coffee and tea) and butyrate-producing bacteria such as cluster XIVa and *Faecalibacterium prausnitzii* [30]. Polyphenols from green tea could also contribute to increasing butyrate producers, with applicability in diverse colonic diseases [31,32].

In previous studies, AhR has been suggested as a mediator of coffee’s effects in the colon, which could explain, in part, the anti-inflammatory effects of this popular beverage [33]. However, AhR antagonists such as epigallocatechingallate (EGCG) and epigallocatechin (EGC) have been detected [34] and associated with the anticancer activity of green tea [35]. During the 48 h fermentation conducted with coffee and green tea, both treatments caused a metabolic production able to induce significant activation of the AhR (see Figure 5). This could be a positive outcome due to the multiple beneficial functions of AhR in the intestinal context that include immune regulation, tissue repair, and homeostasis [36,37,38].

## 5. Conclusions

Among the advantages of batch fermentation experiments are their simplicity, flexibility, adaptability, and cost-effectiveness. However, most existing models simulating the gut microbiota in batches only consider the luminal ecosystem. Thus, the inclusion of mucin-covered microcosms presented in this work to simulate the gut mucosal microbiota could contribute to a more accurate representation of the intestinal ecosystem in short-term static experiments. The applicability of the set-up proposed was demonstrated in the study of two commonly consumed beverages, but it can be extended to a wide spectrum of substrates.

## Figures and Tables

**Table 1:** Results of the UPLC quantification of ammonia and biogenic amines by the end of the fermentation in the M-batches (48 h).

Metabolites	Control (Tea M-Batches)	Tea Treatment	Control (Coffee M-Batches)	Coffee Treatment
Ammonia (mM)	7.80 ± 2.10	9.89 ± 0.63	1.83 ± 0.36	2.11 ± 0.12
2-phenylethylamine(μM)	36.17 ± 3.74	42.29 ± 1.94	ND	ND
Tyramine (μM)	12.68 ± 2.86	18.01 ± 0.42	ND	ND
Putrescine (μM)	531.99 ± 5.59	517.18 ± 0.52	ND	ND
Cadaverine (μM)	523.5 ± 54.11	443.7 ± 5.71	15.83 ± 0.36	15.74 ± 0.34

Data are shown as mean ± SEM. Statistical comparisons were performed using an unpaired *t*-test, and no significant differences were identified between the control and treatment experiments. ND: not detected.

## Data Availability

Data are contained within the article and Supplementary Materials.

## Supplementary Materials

The following supporting information can be downloaded at: https://www.mdpi.com/article/10.3390/microorganisms12020236/s1, which includes: Supplementary Table S1: Target bacteria population measured by qPCR, protocols and annealing temperatures validated [39,40,41,42,43,44,45,46,47,48]. Supplementary Table S2: Phylogenetic analysis using 16S rRNA sequencing for the inoculum A (pool of feces) used to perform the green tea M-batches experiment. Supplementary Table S3: Phylogenetic analysis using 16S rRNA sequencing for the inoculum B (individual donor) used to perform the coffee M-batches experiment. Supplementary Figure S1: Phylum level frequency analysis for the inocula used to perform the M-batch experiments.
