# Supplementary material for: M-Batches to Simulate Luminal and Mucosal Human Gut Microbial Ecosystems: A Case Study of the Effects of Coffee and Green Tea
Source: Microorganisms. 2024 Jan 23;12(2):236. doi: 10.3390/microorganisms12020236 (PMC10891782; doi:10.3390/microorganisms12020236)

Supplementary Material

microorganisms

Article

M-Batches to Simulate Luminal and Mucosal Human Gut Microbial Ecosystems: A Case Study of the Effects of Coffee and Green Tea

Elizabeth Goya-Jorge [1,2,†], Irma Gonza [1,†], Caroline Douny [3], Marie-Louise Scippo [3] and Véronique Delcenserie [1,*]

1 Laboratory of Food Quality Management, Department of Food Sciences, FARAH-Veterinary Public Health, University of Liège, B43b, 4000 Liège, Belgium; egoya@uliege.be (E.G.-J.); iegonza@uliege.be (I.G.)
2 Intestinal Regenerative Medicine Laboratory, Department of Clinical Sciences, College of Veterinary Medicine, North Carolina State University, Raleigh, NC 27606, USA
3 Laboratory of Food Analysis, Department of Food Sciences, FARAH-Veterinary Public Health, University of Liège, B43b, 4000 Liège, Belgium; cdouny@uliege.be (C.D.); mlscippo@uliege.be (M.-L.S.)
* Correspondence: veronique.delcenserie@uliege.be; Tel.: +32-4366-5124
† These authors contributed equally to this work.

Supplementary Table S1. Target bacteria population measured by qPCR, protocols and annealing temperatures validated.

Target	Tann[a] (ºC)	[nM][b]	Validated Protocol	References (Sequences)
Total bacteria	61.5	500	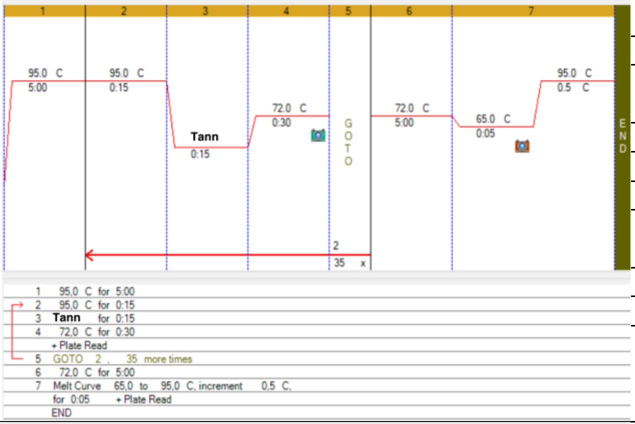	[1]
Cluster XIVa	61.5	500		[2]
Cluster IV	61.5	500		
Roseburia spp.	61.5	500		[3]
Bacteroides-Prevotella spp.	54.0	500		[4]
Bifidobacterium spp.	54.0	500		[5]
Lactobacilli	58.0	300		[6]
Veillonella spp.	58.0	500		
Akkermansia muciniphila	54.0	500		[7]
Faecalibacterium prausnitzii	54.0	500		[4,8]
Phascolarctobacterium faecium	58.0	150		[9]
Coprococcus spp.	63.0	800	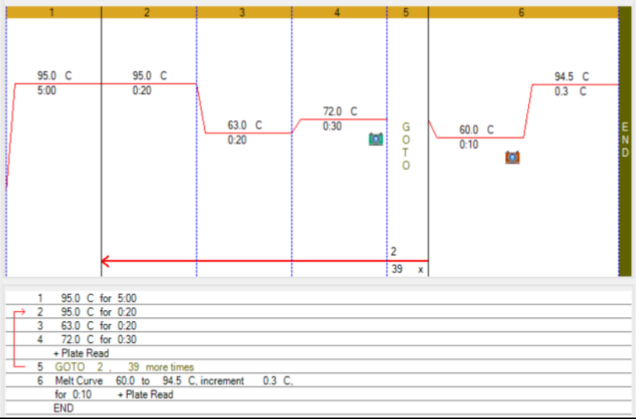	[10]

[a] Tann: Temperature of annealing, [b] Concentration of primer oligosaccharides (forward and reverse) expressed in nM.

REFERENCES

1. Bacchetti De Gregoris, T.; Aldred, N.; Clare, A.S.; Burgess, J.G. Improvement of Phylum- and Class-Specific Primers for Real-Time PCR Quantification of Bacterial Taxa. *J Microbiol Methods* **2011**, *86*, 351–356, doi:10.1016/j.mimet.2011.06.010.
2. Matsuki, T.; Watanabe, K.; Fujimoto, J.; Takada, T.; Tanaka, R. Use of 16S RRNA Gene-Targeted Group-Specific Primers for Real-Time PCR Analysis of Predominant Bacteria in Human Feces. *Appl Environ Microbiol* **2004**, *70*, 7220–7228, doi:10.1128/AEM.70.12.7220-7228.2004.
3. Ramirez-Farias, C.; Slezak, K.; Fuller, Z.; Duncan, A.; Holtrop, G.; Louis, P. Effect of Inulin on the Human Gut Microbiota: Stimulation of Bifidobacterium Adolescentis and Faecalibacterium Prausnitzii. *Br J Nutr* **2009**, *101*, 541–550, doi:10.1017/S0007114508019880.
4. Morel, F.B.; Oozeer, R.; Piloquet, H.; Moyon, T.; Pagniez, A.; Knol, J.; Darmaun, D.; Michel, C. Preweaning Modulation of Intestinal Microbiota by Oligosaccharides or Amoxicillin Can Contribute to Programming of Adult Microbiota in Rats. *Nutrition* **2015**, *31*, 515–522, doi:10.1016/j.nut.2014.09.011.
5. Amit-Romach, E.; Sklan, D.; Uni, Z. Microflora Ecology of the Chicken Intestine Using 16S Ribosomal DNA Primers. *Poult Sci* **2004**, *83*, 1093–1098, doi:10.1093/ps/83.7.1093.
6. Rinttilä, T.; Kassinen, A.; Malinen, E.; Krogius, L.; Palva, A. Development of an Extensive Set of 16S RDNA-Targeted Primers for Quantification of Pathogenic and Indigenous Bacteria in Faecal Samples by Real-Time PCR. *J Appl Microbiol* **2004**, *97*, 1166–1177, doi:10.1111/j.1365-2672.2004.02409.x.
7. Everard, A.; Belzer, C.; Geurts, L.; Ouwerkerk, J.P.; Druart, C.; Bindels, L.B.; Guiot, Y.; Derrien, M.; Muccioli, G.G.; Delzenne, N.M.; et al. Cross-Talk between Akkermansia Muciniphila and Intestinal Epithelium Controls Diet-Induced Obesity. *Proceedings of the National Academy of Sciences* **2013**, *110*, 9066–9071, doi:10.1073/pnas.1219451110.
8. Wang, R.F.; Cao, W.W.; Cerniglia, C.E. PCR Detection and Quantitation of Predominant Anaerobic Bacteria in Human and Animal Fecal Samples. *Appl Environ Microbiol* **1996**, *62*, 1242–1247, doi:10.1128/aem.62.4.1242-1247.1996.
9. Wu, F.; Guo, X.; Zhang, J.; Zhang, M.; Ou, Z.; Peng, Y. Phascolarctobacterium Faecium Abundant Colonization in Human Gastrointestinal Tract. *Exp Ther Med* **2017**, *14*, 3122–3126, doi:10.3892/etm.2017.4878.
10. Lyra, A.; Krogius-Kurikka, L.; Nikkilä, J.; Malinen, E.; Kajander, K.; Kurikka, K.; Korpela, R.; Palva, A. Effect of a Multispecies Probiotic Supplement on Quantity of Irritable Bowel Syndrome-Related Intestinal Microbial Phylotypes. *BMC Gastroenterol* **2010**, *10*, 110, doi:10.1186/1471-230X-10-110.

Supplementary Table S2. Phylogenetic analysis using 16S rRNA sequencing for the Inoculum A (pool of feces) used to perform the green tea M-batches experiment

Kingdom	Phylum	Class	Order	Family	Genus	OTUs Pool of feces
Bacteria	Actinomycetota	Actinomycetes	Bifidobacteriales	Bifidobacteriaceae	*Bifidobacterium*	8
Bacteria	Actinomycetota	Coriobacteriia	Coriobacteriales	Coriobacteriales_fa	*Coriobacteriales*_fa_ge	2
Bacteria	Actinomycetota	Coriobacteriia	Coriobacteriales	Coriobacteriales_fa	*Coriobacteriales*_ge_ge	1
Bacteria	Actinomycetota	Coriobacteriia	Eggerthellales	Eggerthellaceae	*Eggerthella*	1
Bacteria	Bacillota	Erysipelotrichia	Erysipelotrichales	Coprobacillaceae	*Catenibacterium*	8
Bacteria	Bacillota	Erysipelotrichia	Erysipelotrichales	Coprobacillaceae	*Erysipelatoclostridiaceae*_ge	6
Bacteria	Bacillota	Erysipelotrichia	Erysipelotrichales	Coprobacillaceae	*Thomasclavelia*	1
Bacteria	Bacillota	Erysipelotrichia	Erysipelotrichales	Coprobacillaceae	*Erysipelotrichaceae*_UCG-003	3
Bacteria	Bacillota	Erysipelotrichia	Erysipelotrichales	Erysipelotrichaceae	*Erysipelotrichaceae*_ge	7
Bacteria	Bacillota	Erysipelotrichia	Erysipelotrichales	Erysipelotrichaceae	*Holdemanella*	44
Bacteria	Bacillota	Erysipelotrichia	Erysipelotrichales	Erysipelotrichaceae	*Holdemania*	2
Bacteria	Bacillota	Erysipelotrichia	Erysipelotrichales	Erysipelotrichaceae	*Merdibacter*	2
Bacteria	Bacillota	Bacilli	Lactobacillales	Enterococcaceae	*Enterococcus*	1
Bacteria	Bacillota	Bacilli	Lactobacillales	Lactobacillaceae	*Pediococcus*	1
Bacteria	Bacillota	Bacilli	Lactobacillales	Streptococcaceae	*Lactococcus*	2
Bacteria	Bacillota	Bacilli	Lactobacillales	Streptococcaceae	*Streptococcus*	12
Bacteria	Bacillota	Bacilli	RF39	RF39_fa	RF39_ge	8
Bacteria	Bacillota	Clostridia	Eubacteriales	Christensenellaceae	*Christensenellaceae*_ge	9
Bacteria	Bacillota	Clostridia	Eubacteriales	Christensenellaceae	*Christensenellaceae*_R-7_group	118
Bacteria	Bacillota	Clostridia	Clostridia_or	Clostridia_fa	*Clostridia*_ge	23
Bacteria	Bacillota	Clostridia	Clostridia_UCG-014	Clostridia_UCG-014_fa	*Clostridia*_UCG-014_ge	55
Bacteria	Bacillota	Clostridia	Clostridia_vadinBB60_group	Clostridia_vadinBB60_group_fa	*Clostridia_vadinBB60*_group_ge	37
Bacteria	Bacillota	Clostridia	Eubacteriales	Clostridiaceae	*Clostridium_sensu_stricto_1*	7
Bacteria	Bacillota	Clostridia	Eubacteriales	Defluviitaleaceae	*Defluviitaleaceae*_UCG-011	1
Bacteria	Bacillota	Clostridia	Eubacteriales	Lachnospiraceae	*Agathobacter*	112
Bacteria	Bacillota	Clostridia	Eubacteriales	Lachnospiraceae	*Anaerostipes*	8
Bacteria	Bacillota	Clostridia	Eubacteriales	Lachnospiraceae	*Blautia*	84
Bacteria	Bacillota	Clostridia	Eubacteriales	Lachnospiraceae	CAG-56	5
Bacteria	Bacillota	Clostridia	Eubacteriales	Lachnospiraceae	*Coprococcus*	544

Bacteria	Bacillota	Clostridia	Eubacteriales	Lachnospiraceae	*Cuneatibacter*	1
Bacteria	Bacillota	Clostridia	Eubacteriales	Lachnospiraceae	*Dorea*	90
Bacteria	Bacillota	Clostridia	Eubacteriales	Lachnospiraceae	*Fusicatenibacter*	48
Bacteria	Bacillota	Clostridia	Eubacteriales	Lachnospiraceae	*Lachnoclostridium*	92
Bacteria	Bacillota	Clostridia	Eubacteriales	Lachnospiraceae	*Lachnospira*	119
Bacteria	Bacillota	Clostridia	Eubacteriales	Lachnospiraceae	*Lachnospiraceae*_FCS020_group	2
Bacteria	Bacillota	Clostridia	Eubacteriales	Lachnospiraceae	*Lachnospiraceae*_ge	935
Bacteria	Bacillota	Clostridia	Eubacteriales	Lachnospiraceae	*Lachnospiraceae*_ND3007_group	2
Bacteria	Bacillota	Clostridia	Eubacteriales	Lachnospiraceae	*Lachnospiraceae*_NK4A136_group	368
Bacteria	Bacillota	Clostridia	Eubacteriales	Lachnospiraceae	*Lachnospiraceae*_NK4B4_group	1
Bacteria	Bacillota	Clostridia	Eubacteriales	Lachnospiraceae	*Lachnospiraceae*_UCG-001	13
Bacteria	Bacillota	Clostridia	Eubacteriales	Lachnospiraceae	*Lachnospiraceae*_UCG-004	53
Bacteria	Bacillota	Clostridia	Eubacteriales	Lachnospiraceae	*Lachnospiraceae*_UCG-010	3
Bacteria	Bacillota	Clostridia	Eubacteriales	Lachnospiraceae	*Marvinbryantia*	2
Bacteria	Bacillota	Clostridia	Eubacteriales	Lachnospiraceae	*Roseburia*	48
Bacteria	Bacillota	Clostridia	Eubacteriales	Lachnospiraceae	*Sellimonas*	1
Bacteria	Bacillota	Clostridia	Eubacteriales	Lachnospiraceae	*Lachnospirales*_ge	1
Bacteria	Bacillota	Clostridia	Eubacteriales	Oscillospiraceae	*Monoglobus*	38
Bacteria	Bacillota	Clostridia	Eubacteriales	Clostridiaceae	*Butyricicoccaceae*_ge	1
Bacteria	Bacillota	Clostridia	Eubacteriales	Clostridiaceae	*Butyricicoccus*	29
Bacteria	Bacillota	Clostridia	Eubacteriales	Oscillospiraceae	*Acetanaerobacterium*	1
Bacteria	Bacillota	Clostridia	Eubacteriales	Oscillospiraceae	*Colidextribacter*	22
Bacteria	Bacillota	Clostridia	Eubacteriales	Oscillospiraceae	*Flavonifractor*	10
Bacteria	Bacillota	Clostridia	Eubacteriales	Oscillospiraceae	*Intestinimonas*	2
Bacteria	Bacillota	Clostridia	Eubacteriales	Oscillospiraceae	NK4A214_group	89
Bacteria	Bacillota	Clostridia	Eubacteriales	Oscillospiraceae	*Oscillibacter*	22
Bacteria	Bacillota	Clostridia	Eubacteriales	Oscillospiraceae	*Oscillospira*	1
Bacteria	Bacillota	Clostridia	Eubacteriales	Oscillospiraceae	*Oscillospiraceae*_ge	30
Bacteria	Bacillota	Clostridia	Eubacteriales	Oscillospiraceae	*Pseudoflavonifractor*	1
Bacteria	Bacillota	Clostridia	Eubacteriales	Oscillospiraceae	UCG-002	269
Bacteria	Bacillota	Clostridia	Eubacteriales	Oscillospiraceae	UCG-003	48
Bacteria	Bacillota	Clostridia	Eubacteriales	Oscillospiraceae	UCG-005	149
Bacteria	Bacillota	Clostridia	Eubacteriales	Oscillospiraceae	V9D2013_group	1
Bacteria	Bacillota	Clostridia	Eubacteriales	Oscillospirales_fa	*Oscillospirales*_ge	177
Bacteria	Bacillota	Clostridia	Eubacteriales	Oscillospiraceae	*Anaerofilum*	2

Bacteria	Bacillota	Clostridia	Eubacteriales	Oscillospiraceae	CAG-352	4
Bacteria	Bacillota	Clostridia	Eubacteriales	Oscillospiraceae	*Candidatus_Soleaferrea*	1
Bacteria	Bacillota	Clostridia	Eubacteriales	Oscillospiraceae	DTU089	4
Bacteria	Bacillota	Clostridia	Eubacteriales	Oscillospiraceae	*Faecalibacterium*	838
Bacteria	Bacillota	Clostridia	Eubacteriales	Oscillospiraceae	*Fournierella*	14
Bacteria	Bacillota	Clostridia	Eubacteriales	Oscillospiraceae	*Negativibacillus*	4
Bacteria	Bacillota	Clostridia	Eubacteriales	Oscillospiraceae	*Phocea*	2
Bacteria	Bacillota	Clostridia	Eubacteriales	Oscillospiraceae	*Ruminococcaceae*_ge	162
Bacteria	Bacillota	Clostridia	Eubacteriales	Oscillospiraceae	*Ruminococcus*	321
Bacteria	Bacillota	Clostridia	Eubacteriales	Oscillospiraceae	*Subdoligranulum*	599
Bacteria	Bacillota	Clostridia	Eubacteriales	Oscillospiraceae	UBA1819	10
Bacteria	Bacillota	Clostridia	Eubacteriales	UCG-010	UCG-010_ge	83
Bacteria	Bacillota	Clostridia	Peptostreptococcales	Anaerovoracaceae	*Anaerovoracaceae*_Family_XIII_AD3011_group	13
Bacteria	Bacillota	Clostridia	Peptostreptococcales	Anaerovoracaceae	*Anaerovoracaceae*_Family_XIII_UCG-001	11
Bacteria	Bacillota	Clostridia	Peptostreptococcales	Anaerovoracaceae	*Anaerovoracaceae*_ge	5
Bacteria	Bacillota	Tissierellia	Tissierellales	Peptoniphilaceae	*Peptoniphilus*	1
Bacteria	Bacillota	Clostridia	Eubacteriales	Peptostreptococcaceae	*Intestinibacter*	5
Bacteria	Bacillota	Clostridia	Eubacteriales	Peptostreptococcaceae	*Romboutsia*	10
Bacteria	Bacillota	Clostridia	Eubacteriales	Peptostreptococcaceae	*Terrisporobacter*	2
Bacteria	Bacillota	Negativicutes	Acidaminococcales	Acidaminococcaceae	*Phascolarctobacterium*	125
Bacteria	Bacillota	Negativicutes	Selenomonadales	Selenomonadaceae	*Megamonas*	1
Bacteria	Bacillota	Negativicutes	Selenomonadales	Selenomonadaceae	*Mitsuokella*	3
Bacteria	Bacillota	Negativicutes	Veillonellales	Veillonellaceae	*Dialister*	311
Bacteria	Bacillota	Negativicutes	Veillonellales	Veillonellaceae	*Megasphaera*	16
Bacteria	Bacillota	Negativicutes	Veillonellales	Veillonellaceae	*Veillonella*	2
Bacteria	Bacillota	Negativicutes	Veillonellales	Veillonellaceae	*Veillonellaceae*_ge	4
Bacteria	Bacteria_ph	Bacteria_cl	Bacteria_or	Bacteria_fa	*Bacteria*_ge	7
Bacteria	Bacteroidota	Bacteroidia	Bacteroidales	Bacteroidaceae	*Bacteroides*	753
Bacteria	Bacteroidota	Bacteroidia	Bacteroidales	Bacteroidales_fa	*Bacteroidales*_ge	42
Bacteria	Bacteroidota	Bacteroidia	Bacteroidales	Barnesiellaceae	*Barnesiella*	109
Bacteria	Bacteroidota	Bacteroidia	Bacteroidales	Barnesiellaceae	*Coprobacter*	4
Bacteria	Bacteroidota	Bacteroidia	Bacteroidales	Odoribacteraceae	*Butyricimonas*	25
Bacteria	Bacteroidota	Bacteroidia	Bacteroidales	Odoribacteraceae	*Odoribacter*	48

Bacteria	Bacteroidota	Bacteroidia	Bacteroidales	Muribaculaceae	CAG-873	3
Bacteria	Bacteroidota	Bacteroidia	Bacteroidales	Prevotellaceae	*Paraprevotella*	14
Bacteria	Bacteroidota	Bacteroidia	Bacteroidales	Prevotellaceae	*Prevotella*	1
Bacteria	Bacteroidota	Bacteroidia	Bacteroidales	Prevotellaceae	*Prevotella*_7	3
Bacteria	Bacteroidota	Bacteroidia	Bacteroidales	Prevotellaceae	*Prevotella*_9	1850
Bacteria	Bacteroidota	Bacteroidia	Bacteroidales	Prevotellaceae	*Prevotellaceae*_ge	1
Bacteria	Bacteroidota	Bacteroidia	Bacteroidales	Prevotellaceae	*Prevotellaceae*_NK3B31_group	38
Bacteria	Bacteroidota	Bacteroidia	Bacteroidales	Rikenellaceae	*Alistipes*	227
Bacteria	Bacteroidota	Bacteroidia	Bacteroidales	Rikenellaceae	*Rikenellaceae*_RC9_gut_group	82
Bacteria	Bacteroidota	Bacteroidia	Bacteroidales	Tannerellaceae	*Parabacteroides*	151
Bacteria	Campylobacterota	Epsilonproteobacteria	Campylobacterales	Helicobacteraceae	*Helicobacter*	2
Bacteria	Cyanobacteriota /Melainacteria	Candidatus Melainabacteria	Candidatus Gastranaerophilales	Candidatus Gastranaerophilaceae	*Gastranaerophilales*_ge	1
Bacteria	Lentisphaerota	Lentisphaeria	Victivallales	vadinBE97	*vadinBE97*_ge	4
Bacteria	Lentisphaerota	Lentisphaeria	Victivallales	Victivallaceae	*Victivallaceae*_ge	2
Bacteria	Lentisphaerota	Lentisphaeria	Victivallales	Victivallaceae	*Victivallis*	26
Bacteria	Mycoplasmatota	Mollicutes	Acholeplasmatales	Acholeplasmataceae	*Anaeroplasma*	16
Bacteria	Mycoplasmatota	Candidatus Izimaplasma	Candidatus Izemoplasmatales	Candidatus Izemoplasmataceae	*Candidatus Izemoplasma*	1
Bacteria	Pseudomonadota	Alphaproteobacteria	Rhodospirillales	Rhodospirillales_fa	*Rhodospirillales*_fa_ge	49
Bacteria	Pseudomonadota	Betaproteobacteria	Burkholderiales	Burkholderiales_fa	*Burkholderiales*_ge	2
Bacteria	Pseudomonadota	Betaproteobacteria	Burkholderiales	Oxalobacteraceae	*Oxalobacter*	6
Bacteria	Pseudomonadota	Betaproteobacteria	Burkholderiales	Sutterellaceae	*Parasutterella*	45
Bacteria	Pseudomonadota	Betaproteobacteria	Burkholderiales	Sutterellaceae	*Sutterella*	27
Bacteria	Pseudomonadota	Gammaproteobacteria	Enterobacterales	Enterobacteriaceae	*Escherichia-Shigella*	4
Bacteria	Pseudomonadota	Gammaproteobacteria	Pasteurellales	Pasteurellaceae	*Haemophilus*	2
Bacteria	Pseudomonadota	Gammaproteobacteria	Pasteurellales	Pasteurellaceae	*Pasteurellaceae*_ge	1
Bacteria	Saccharimonadota	Saccharimonadia	Saccharimonadales	Saccharimonadaceae	*Saccharimonadaceae*_ge	1
Bacteria	Thermodesulfobacteriota	Desulfovibrionia	Desulfovibrionales	Desulfovibrionaceae	*Bilophila*	9
Bacteria	Thermodesulfobacteriota	Desulfovibrionia	Desulfovibrionales	Desulfovibrionaceae	*Desulfovibrio*	8
Bacteria	Thermodesulfobacteriota	Desulfovibrionia	Desulfovibrionales	Desulfovibrionaceae	*Mailhella*	2
Bacteria	Verrucomicrobiota	Opitutae	Puniceicoccales	Coraliomargaritaceae	*Coraliomargarita*	2
Bacteria	Verrucomicrobiota	Verrucomicrobiae	Verrucomicrobiales	Akkermansiaceae	*Akkermansia*	94

Supplementary Table S3. Phylogenetic analysis using 16S rRNA sequencing for the Inoculum B (individual donor) used to perform coffee M-batches experiment

Kingdom	Phylum	Class	Order	Family	Genus	OTUs Single donor
Bacteria	Actinomycetota	Actinomycetes	Bifidobacteriales	Bifidobacteriaceae	*Bifidobacterium*	2
Bacteria	Actinomycetota	Coriobacteriia	Coriobacteriales	Coriobacteriaceae	*Collinsella*	9
Bacteria	Actinomycetota	Coriobacteriia	Coriobacteriales	Coriobacteriales Incertae Sedis	*Coriobacteriales*_ge	6
Bacteria	Actinomycetota	Coriobacteriia	Eggerthellales	Eggerthellaceae	*Adlercreutzia*	1
Bacteria	Bacillota	Erysipelotrichia	Erysipelotrichales	Coprobacillaceae	*Catenibacterium*	4
Bacteria	Bacillota	Erysipelotrichia	Erysipelotrichales	Coprobacillaceae	*Coprobacillus*	22
Bacteria	Bacillota	Erysipelotrichia	Erysipelotrichales	Coprobacillaceae	*Thomasclavelia*	2
Bacteria	Bacillota	Erysipelotrichia	Erysipelotrichales	Erysipelotrichaceae	*Erysipelotrichaceae*_ge	1
Bacteria	Bacillota	Erysipelotrichia	Erysipelotrichales	Erysipelotrichaceae	*Holdemanella*	20
Bacteria	Bacillota	Erysipelotrichia	Erysipelotrichales	Erysipelotrichaceae	*Solobacterium*	1
Bacteria	Bacillota	Bacilli	Lactobacillales	Carnobacteriaceae	*Granulicatella*	1
Bacteria	Bacillota	Bacilli	Lactobacillales	Streptococcaceae	*Lactococcus*	1
Bacteria	Bacillota	Bacilli	Lactobacillales	Streptococcaceae	*Streptococcus*	42
Bacteria	Bacillota	Bacilli	RF39	RF39_fa	RF39_ge	11
Bacteria	Bacillota	Bacilli	Bacillales	Gemellaceae	*Gemella*	1
Bacteria	Bacillota	Clostridia	Eubacteriales	Christensenellaceae	*Christensenellaceae*_ge	2
Bacteria	Bacillota	Clostridia	Eubacteriales	Christensenellaceae	*Christensenellaceae*_R-7_group	265
Bacteria	Bacillota	Clostridia	Clostridia_or	Clostridia_fa	*Clostridia*_ge	13
Bacteria	Bacillota	Clostridia	Clostridia_UCG-014	Clostridia_UCG-014_fa	*Clostridia*_UCG-014_ge	136
Bacteria	Bacillota	Clostridia	Clostridia_vadinBB60_ group	Clostridia_vadinBB60_group _fa	*Clostridia_vadinBB60*_group_ge	5
Bacteria	Bacillota	Clostridia	Eubacteriales	Eubacteriales_fa	*Eubacteriales*_ge	2
Bacteria	Bacillota	Clostridia	Eubacteriales	Defluviitaleaceae	*Defluviitaleaceae*_UCG-011	3
Bacteria	Bacillota	Clostridia	Eubacteriales	Lachnospiraceae	*Anaerostipes*	2
Bacteria	Bacillota	Clostridia	Eubacteriales	Lachnospiraceae	*Blautia*	22
Bacteria	Bacillota	Clostridia	Eubacteriales	Lachnospiraceae	*Catenibacillus*	1
Bacteria	Bacillota	Clostridia	Eubacteriales	Lachnospiraceae	*Coprococcus*	199
Bacteria	Bacillota	Clostridia	Eubacteriales	Lachnospiraceae	*Dorea*	3

Bacteria	Bacillota	Clostridia	Eubacteriales	Lachnospiraceae	*Eisenbergiella*	3
Bacteria	Bacillota	Clostridia	Eubacteriales	Lachnospiraceae	*Fusicatenibacter*	19
Bacteria	Bacillota	Clostridia	Eubacteriales	Lachnospiraceae	GCA-900066575	4
Bacteria	Bacillota	Clostridia	Eubacteriales	Lachnospiraceae	GCA-900066755	1
Bacteria	Bacillota	Clostridia	Eubacteriales	Lachnospiraceae	*Lachnoclostridium*	85
Bacteria	Bacillota	Clostridia	Eubacteriales	Lachnospiraceae	*Lachnospira*	10
Bacteria	Bacillota	Clostridia	Eubacteriales	Lachnospiraceae	*Lachnospiraceae*_ge	137
Bacteria	Bacillota	Clostridia	Eubacteriales	Lachnospiraceae	*Lachnospiraceae*_ND3007_group	1
Bacteria	Bacillota	Clostridia	Eubacteriales	Lachnospiraceae	*Lachnospiraceae*_NK4A136_group	72
Bacteria	Bacillota	Clostridia	Eubacteriales	Lachnospiraceae	*Lachnospiraceae*_UCG-001	6
Bacteria	Bacillota	Clostridia	Eubacteriales	Lachnospiraceae	*Lachnospiraceae*_UCG-002	1
Bacteria	Bacillota	Clostridia	Eubacteriales	Lachnospiraceae	*Lachnospiraceae*_UCG-004	10
Bacteria	Bacillota	Clostridia	Eubacteriales	Lachnospiraceae	*Lachnospiraceae*_UCG-008	1
Bacteria	Bacillota	Clostridia	Eubacteriales	Lachnospiraceae	*Lachnospiraceae*_UCG-010	1
Bacteria	Bacillota	Clostridia	Eubacteriales	Lachnospiraceae	*Marvinbryantia*	3
Bacteria	Bacillota	Clostridia	Eubacteriales	Lachnospiraceae	*Roseburia*	21
Bacteria	Bacillota	Clostridia	Eubacteriales	Lachnospiraceae	UC5-1-2E3	2
Bacteria	Bacillota	Clostridia	Eubacteriales	Lachnospirales_fa	*Lachnospirales*_ge	2
Bacteria	Bacillota	Clostridia	Eubacteriales	Oscillospiraceae	*Monoglobus*	4
Bacteria	Bacillota	Clostridia	Eubacteriales	Clostridiaceae	*Butyricicoccus*	8
Bacteria	Bacillota	Clostridia	Eubacteriales	Butyricicoccaceae	UCG-009	1
Bacteria	Bacillota	Clostridia	Eubacteriales	Oscillospiraceae	*Colidextribacter*	18
Bacteria	Bacillota	Clostridia	Eubacteriales	Oscillospiraceae	*Flavonifractor*	52
Bacteria	Bacillota	Clostridia	Eubacteriales	Oscillospiraceae	*Intestinimonas*	4
Bacteria	Bacillota	Clostridia	Eubacteriales	Oscillospiraceae	NK4A214_group	103
Bacteria	Bacillota	Clostridia	Eubacteriales	Oscillospiraceae	*Oscillibacter*	10
Bacteria	Bacillota	Clostridia	Eubacteriales	Oscillospiraceae	*Oscillospira*	3
Bacteria	Bacillota	Clostridia	Eubacteriales	Oscillospiraceae	*Oscillospiraceae*_ge	82
Bacteria	Bacillota	Clostridia	Eubacteriales	Oscillospiraceae	UCG-002	272
Bacteria	Bacillota	Clostridia	Eubacteriales	Oscillospiraceae	UCG-003	1
Bacteria	Bacillota	Clostridia	Eubacteriales	Oscillospiraceae	UCG-005	144
Bacteria	Bacillota	Clostridia	Eubacteriales	Oscillospiraceae	UCG-007	1
Bacteria	Bacillota	Clostridia	Eubacteriales	Oscillospiraceae	*Hydrogenoanaerobacterium*	2
Bacteria	Bacillota	Clostridia	Eubacteriales	Oscillospirales_fa	*Oscillospirales*_ge	97
Bacteria	Bacillota	Clostridia	Eubacteriales	Oscillospiraceae	*Anaerofilum*	3

Bacteria	Bacillota	Clostridia	Eubacteriales	Oscillospiraceae	*Anaerotruncus*	2
Bacteria	Bacillota	Clostridia	Eubacteriales	Oscillospiraceae	*Angelakisella*	1
Bacteria	Bacillota	Clostridia	Eubacteriales	Oscillospiraceae	CAG-352	72
Bacteria	Bacillota	Clostridia	Eubacteriales	Oscillospiraceae	DTU089	3
Bacteria	Bacillota	Clostridia	Eubacteriales	Oscillospiraceae	*Faecalibacterium*	405
Bacteria	Bacillota	Clostridia	Eubacteriales	Oscillospiraceae	*Fournierella*	2
Bacteria	Bacillota	Clostridia	Eubacteriales	Oscillospiraceae	*Negativibacillus*	3
Bacteria	Bacillota	Clostridia	Eubacteriales	Oscillospiraceae	*Phocea*	2
Bacteria	Bacillota	Clostridia	Eubacteriales	Oscillospiraceae	*Ruminococcaceae*_ge	94
Bacteria	Bacillota	Clostridia	Eubacteriales	Oscillospiraceae	*Ruminococcus*	122
Bacteria	Bacillota	Clostridia	Eubacteriales	Oscillospiraceae	*Subdoligranulum*	384
Bacteria	Bacillota	Clostridia	Eubacteriales	Oscillospiraceae	UBA1819	30
Bacteria	Bacillota	Clostridia	Eubacteriales	UCG-010	UCG-010_ge	37
Bacteria	Bacillota	Clostridia	Eubacteriales	Peptococcaceae	*Peptococcaceae*_ge	3
Bacteria	Bacillota	Clostridia	Peptostreptococcales	Anaerovoracaceae	*Anaerovoracaceae*_Family_XIII_AD3011_group	3
Bacteria	Bacillota	Clostridia	Peptostreptococcales	Anaerovoracaceae	*Anaerovoracaceae*_Family_XIII_UCG-001	3
Bacteria	Bacillota	Clostridia	Peptostreptococcales	Anaerovoracaceae	*Anaerovoracaceae*_ge	1
Bacteria	Bacillota	Clostridia	Eubacteriales	Peptostreptococcaceae	*Romboutsia*	1
Bacteria	Bacillota	Tissierellia	Tissierellales	Peptoniphilaceae	*Anaerococcus*	1
Bacteria	Bacillota	Tissierellia	Tissierellales	Peptoniphilaceae	*Ezakiella*	1
Bacteria	Bacillota	Clostridia	Eubacteriales	Eubacteriales_fa	*Fenollaria*	2
Bacteria	Bacillota	Tissierellia	Tissierellales	Peptoniphilaceae	*Peptoniphilus*	7
Bacteria	Bacillota	Bacillota_cl	Bacillota_or	Bacillota_fa	*Bacillota*_ge	4
Bacteria	Bacillota	Negativicutes	Acidaminococcales	Acidaminococcaceae	*Phascolarctobacterium*	42
Bacteria	Bacillota	Negativicutes	Veillonellales	Veillonellaceae	*Dialister*	2
Bacteria	Bacillota	Negativicutes	Veillonellales	Veillonellaceae	*Veillonella*	1
Bacteria	Bacteria_ph	Bacteria_cl	Bacteria_or	Bacteria_fa	*Bacteria*_ge	10
Bacteria	Bacteroidota	Bacteroidia	Bacteroidales	Bacteroidaceae	*Bacteroides*	621
Bacteria	Bacteroidota	Bacteroidia	Bacteroidales	Bacteroidales_fa	*Bacteroidales*_ge	8
Bacteria	Bacteroidota	Bacteroidia	Bacteroidales	Barnesiellaceae	*Barnesiella*	2
Bacteria	Bacteroidota	Bacteroidia	Bacteroidales	Barnesiellaceae	*Coprobacter*	9
Bacteria	Bacteroidota	Bacteroidia	Bacteroidales	Odoribacteraceae	*Butyricimonas*	2
Bacteria	Bacteroidota	Bacteroidia	Bacteroidales	Odoribacteraceae	*Odoribacter*	6

Bacteria	Bacteroidota	Bacteroidia	Bacteroidales	Porphyromonadaceae	*Sanguibacteroides*	1
Bacteria	Bacteroidota	Bacteroidia	Bacteroidales	Porphyromonadaceae	*Porphyromonas*	7
Bacteria	Bacteroidota	Bacteroidia	Bacteroidales	Prevotellaceae	*Paraprevotella*	10
Bacteria	Bacteroidota	Bacteroidia	Bacteroidales	Prevotellaceae	*Prevotella*	16
Bacteria	Bacteroidota	Bacteroidia	Bacteroidales	Prevotellaceae	*Prevotellaceae*_ge	2
Bacteria	Bacteroidota	Bacteroidia	Bacteroidales	Prevotellaceae	*Prevotellaceae*_NK3B31_group	149
Bacteria	Bacteroidota	Bacteroidia	Bacteroidales	Prevotellaceae	*Prevotellaceae*_UCG-001	18
Bacteria	Bacteroidota	Bacteroidia	Bacteroidales	Rikenellaceae	*Alistipes*	128
Bacteria	Bacteroidota	Bacteroidia	Bacteroidales	Rikenellaceae	*Rikenellaceae*_RC9_gut_group	24
Bacteria	Bacteroidota	Bacteroidia	Bacteroidales	Tannerellaceae	*Parabacteroides*	46
Bacteria	Bacteroidota	Bacteroidia	Bacteroidia_or	Bacteroidia_fa	*Bacteroidia*_ge	3
Bacteria	Cyanobacteriota /Melainacteria	Candidatus Melainabacteria	Candidatus Gastranaerophilales	Candidatus Gastranaerophilaceae	*Gastranaerophilales*_ge	2
Bacteria	Elusimicrobiota	Elusimicrobia	Elusimicrobiales	Elusimicrobiaceae	*Elusimicrobium*	2
Bacteria	Fusobacteriota	Fusobacteriia	Fusobacteriales	Fusobacteriaceae	*Fusobacterium*	1
Bacteria	Lentisphaerota	Lentisphaeria	Victivallales	Victivallaceae	*Victivallis*	1
Bacteria	Pseudomonadota	Betaproteobacteria	Burkholderiales	Oxalobacteraceae	*Oxalobacter*	1
Bacteria	Pseudomonadota	Betaproteobacteria	Burkholderiales	Sutterellaceae	*Parasutterella*	5
Bacteria	Pseudomonadota	Betaproteobacteria	Burkholderiales	Sutterellaceae	*Sutterella*	10
Bacteria	Pseudomonadota	Gammaproteobacteria	Enterobacterales	Enterobacterales_fa	*Enterobacterales*_ge	1
Bacteria	Pseudomonadota	Gammaproteobacteria	Enterobacterales	Enterobacteriaceae	*Escherichia-Shigella*	6
Bacteria	Pseudomonadota	Alphaproteobacteria	Hyphomicrobiales	Hyphomicrobiales_fa	*Enhydrobacter*	1
Bacteria	Thermodesulfobacteriota	Desulfovibrionia	Desulfovibrionales	Desulfovibrionaceae	*Bilophila*	4
Bacteria	Thermodesulfobacteriota	Desulfovibrionia	Desulfovibrionales	Desulfovibrionaceae	*Desulfovibrionaceae*_ge	33
Bacteria	Verrucomicrobiota	Verrucomicrobiae	Verrucomicrobiales	Akkermansiaceae	*Akkermansia*	5682

Supplementary Figure S1. Phylum level frequency analysis for the inocula used to perform the M-batch experiments

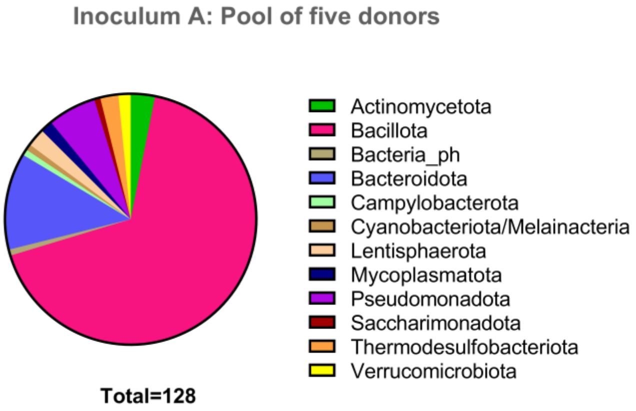

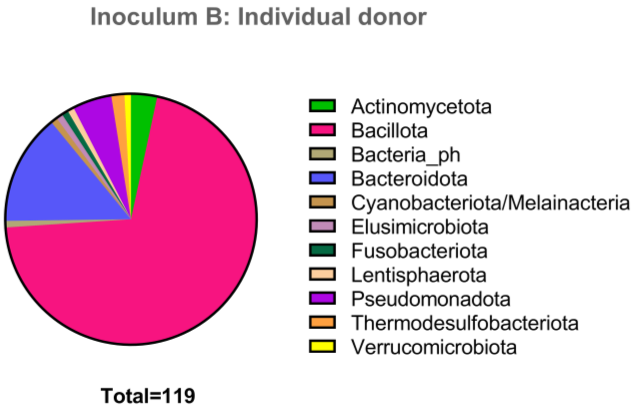

Supplement: Supplementary file 1 [file microorganisms-12-00236-s001.zip › microorganisms-2789769-supplementary.pdf]
